# Small Pox—Vaccination—Varioloid

**Published:** 1854-03

**Authors:** 


### SMALL POX—VACCINATION—VARIOLOID.

For the week ending Feb. 11, 1854, in New York city, fifty-seven persons died of Small Pox, making two hundred and forty-five deaths, or nearly six deaths a day from that terrible disease since the 1st of January last. Vaccination is the only protection, except that of having it in the natural way. Vaccination is performed at all the Dispensaries, *free of charge.* I earnestly advise the permanent residents of New York to apply to some well known, long established, and reputable physician, whose duty it is to have always on hand fresh vaccine virus, taken by them from patients known to themselves personally to be young, healthful, and of sound constitution and blood,—otherwise Vaccination cannot be relied on as a preventive against Small Pox.

What is Vaccination? What is Varioloid? How often ought Vaccination to be repeated? How may I know for myself whether Vaccination has indisputably taken? A satisfactory answer may be had by purchasing the February number of Dixon's "Scalpel," for 25 cents, from Dewitt and Davenport, Tribune Buildings, New York; Peterson, Philadelphia; Reading & Co., Boston. There are two other admirable editorial articles in this same number, which are of vital interest to the whole community—What is a Catarrh or common Cold? What is Dyspepsia?

### WHEN DOES VACCINATION TAKE?

On the seventh or eighth day after the "operation," we see a brown centre, of an oval shape, surrounded by pearl colored dots; outside of these is a reddish appearance, fading away into the natural color of the skin; after the vesicle has passed away, which is in seven or eight days more, a perfect vaccine crust or scab presents itself in the place of the vesicle, so that it cannot be certainly told whether a vaccination has "taken" until the fourteenth or fifteenth day. When the pearly dots do not appear then it has not taken, and the person is either unprotected, or a previous vaccination has not run out. If after three trials no pearly dots appear around any sore which may present itself, then we may feel assured that the person remains fully protected.

